# Congenital Deformity of the Superior Extremity

**Published:** 1879-02

**Authors:** 


Congenital Deformity of the Superior Extremity.—In the Vienna section of the Association of Physicians of Lower Austria, F. V. Becker presented two cases of congenital arrest of development. The patients, brother and sister, belong to a poor family. Father and mother are healthy; three children have died, and presented no abnormal development. Of the living children, one of them (a girl) is normally constituted; the other three are deformed, as described below:

The boy is well developed, but delicate, and badly nourished. Both superior extremities are very much shortened. On the left side this shortening is due to a total absence of the bones of the forearm. On the right side it is due to shortening of the humerus, and to the fact that radius and ulna are merged in one bone of about one-third of the normal length. The shoulder joint is defective both in development and in width. On the left side the elbow joint is wanting; on the right side its motion is limited, reminding one of partial anchylosis. We find only rudiments of the metacarpal bones of the thumbs, and these rudiments are firmly united to the first and second phalanx of the index finger.

In the girl these deformities of the thumbs are less marked, and the superior extremities are of normal length.

In both children, especially in the boy, we find enormous enlargement of the heart dependent upon stenosis aortæ, easily recognized. We cannot determine whether there is insufficiency or not. Careful examination fails to reveal a radial pulse, and the absence of pulsation in the fossa clavicularis suggests the absence, or, at least, an abnormal course, of the subclavia. Von Becker considers the abnormal condition of the heart, or, at least, the stenosis of the aorta as the primary affection, and that the arrest of development was due to an insufficient supply of blood to the affected organs during intra-uterine life. This explanation has already been accepted by various authors, but probably nowhere, in our literature, do we find examples that demonstrate this fact so plainly as the cases here presented.—*St. Louis Courier of Medicine.*

